# Informal Meeting of Physicians

**Published:** 1862-11

**Authors:** 


*Informal Meeting of Physicians.*—At an informal meeting of the physicians of Buffalo, held at the rooms of the Medical Association, Dr. T. T. Lockwood was called to the Chair and Julius F. Miner appointed Secretary.

After a brief explanation of the objects of the meeting by Dr. Wyckoff, Dr. Eastman presented the following resolutions, which were unanimously adopted:

*Resolved*, That, for the purpose of showing our estimation of the worth and respect for the memory of Lieut. Mackay, and of extending our sympathy to the family and friends of the deceased, we attend the funeral in a body and wear the usual badge of mourning.

*Resolved*, That the Secretary communicate this action to Dr. Mackay and family, and make such other notice of it as he may think proper.

